# Sciatic-nerve radiofrequency ablation for phantom limb pain: A case report

**DOI:** 10.1016/j.inpm.2024.100388

**Published:** 2024-01-26

**Authors:** Melissa Schwartz, Pranamya Suri, Scott Benkovic, Eric Muneio, Nikhil Gopal, Akhil Chhatre

**Affiliations:** a Department of Physical Medicine & Rehabilitation, Johns Hopkins Hospital, 1800 Orleans St, Baltimore, MD, 21287, USA; b Department of Transitional Medicine, Detroit Medical Center Sinai-Grace Hospital, 6071 Outer Dr W, Detroit, MI, 48235, USA

## Abstract

Phantom and residual limb pain are commonly experienced by the majority of amputees, and are often difficult to treat not adequately relieved with medical treatment alone. While extensively studied, the pathophysiology of refractory pain is still unclear, with many proposed mechanisms under investigation (Wilkes et al., 2008). Limited existing literature suggests percutaneous interventions including radiofrequency ablation (RFA) may be promising treatment modalities for patients who have pain refractory to oral agents (Sperry et al., 2023). We present a patient with persistent phantom limb and cancer-associated acetabular pain following hip disarticulation who underwent sciatic-notch RFA for pain management.

## Abbreviation list

AP: Anteroposterior
HD: Hip Disarticulation
LEA: Lower Extremity Amputation
PLP: Phantom Limb Pain
RLP: Residual Limb Pain
RFA: Radiofrequency Ablation
US: United States

## 1 Introduction

Nearly 2 million people in the United States alone are living with limb loss based on available survey data, and existing literature suggests the prevalence continues to increase with estimates of 300-500 amputations occurring daily [5,15]. While many advancements in surgical interventions and care as well as preventive measures have been implemented aiming to reduce peripheral vascular and diabetic limb loss, the increased size of the aging population with these health conditions and stable rates of congenital, traumatic or cancer-related amputations contribute to the rising rate in today's population [6,15,16].

Of the amputation types noted in existing literature, lower extremity amputations are most common, with an estimated 150,000 patients undergoing lower limb amputation in the US each year [4]. They are most often associated with diabetes and vascular disease, with around 16 % in the setting of trauma, and less than one-percent which are cancer-related. Of these lower extremity amputations, hip-disarticulation is much less common, accounting for around 0.5 % of lower extremity amputations on a yearly basis [7].

Regardless of the etiology requiring lower-extremity amputation, many patients subsequently experience severe post-operative pain, encompassed by residual limb and phantom limb pain. Phantom limb pain (PLP) is defined as a variety of pain types the patient experiences in any part of the amputated limb. Often variable in severity and frequency, PLP is described as stinging, cramping, sharp, stabbing, burning or shooting, affecting up to 50-80 % of patients at some point in the post-operative period [2,8]. Pain is typically experienced within the first month of following the procedure and is reported moderately diminish over 6 months, however limited prospective research suggests persistence in some form beyond 6 months in anywhere from 10 to 85 % of patients [4,8,17,18]^,^. While a common phenomenon, the pathophysiology of PLP is not yet entirely understood [2,8]. Existing literature explores both central and peripheral factors that may contribute to pain. These include cortical somatosensory remapping as well as peripheral ectopia from spontaneous firing of peripheral nerves [8,9]. However no clear mechanism has yet been defined.

Given the mechanisms underlying phantom pain are still under investigation, treatment remains challenging. Many modalities of treatment exist, including oral neuropathic pain agents, physical therapy, acupuncture, neuromodulation and others [2,10]. However, many patients report inadequate relief with these treatments alone. Limited existing literature suggests percutaneous interventions including radiofrequency ablation (RFA) may be a promising treatment modality for patients who have pain refractory to oral agents [1]. We present a patient with persistent phantom limb and cancer-associated acetabular pain following hip disarticulation who underwent sciatic-nerve RFA at the sciatic notch for pain management.

## 2 Case

A 73-year-old female with a past medical history of hypertension, anemia, stage IV leiomyosarcoma of the left thigh status-post left hip disarticulation in 2021, and a persistent mass encasing the left acetabulum, initially presented with worsening left buttock and phantom limb pain after initiation of radiation treatment to the residual limb in September 2022. She was initially evaluated by our service March of 2023 for ongoing symptoms prior to undergoing the procedure in July of 2023.

**Fig. 1:**
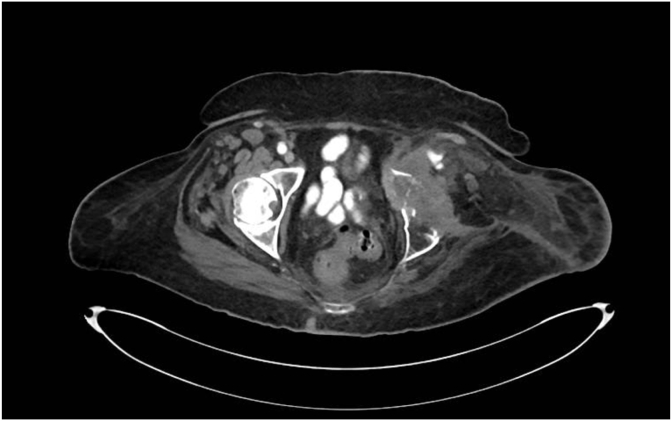
Axial CT pelvis demonstrating enlarging left acetabular mass encasing the left external iliac artery and involving the left sciatic nerve.

The patient's pain was described as sharp and aching, in addition to an electric sensation located at the amputated left ankle and knee. She experienced the pain on daily basis with worsening symptoms at night resulting in significant sleep disruption. She was unable to identify any provoking factors, but did report a need for frequent repositioning while seated. Repeat imaging including CT abdomen/pelvis with contrast completed in April 2023, demonstrated an enlarging left acetabular mass encasing the left external iliac artery and involving the left sciatic nerve (Fig. 1). She was followed by an outpatient pain management specialist, however, her symptoms were refractory to increasing doses of topical analgesics, oral opioid agonists, antispasmodics, oral neuropathic pain relief agents, as well as physical therapy. The patient therefore opted to undergo evaluation for RFA of the sciatic nerve for improved and sustained pain relief.

## 3 Methods

**Fig. 2:**
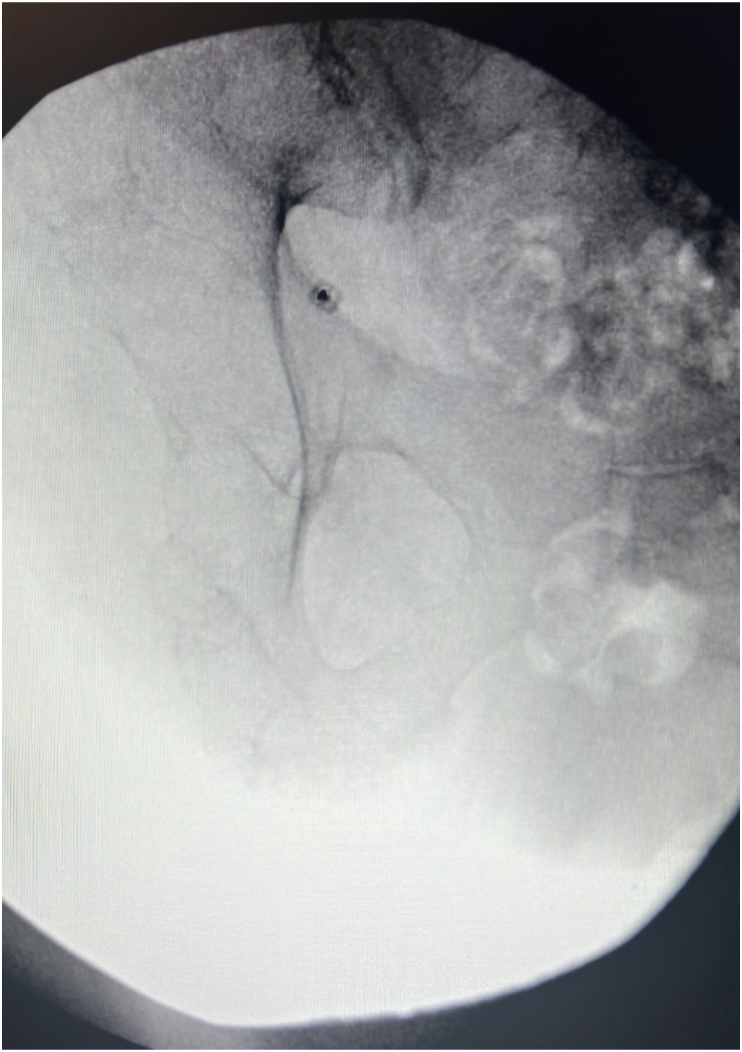
Left sciatic nerve block at the sciatic notch.

The patient initially underwent regional block of the left sciatic nerve. Prior to the procedure, the patient was placed in a prone position, and the area was prepped in a sterile manner. She received local anesthetic with 1 % preservative free lidocaine to the skin. Regional block was completed under fluoroscopic guidance. The fluoroscope was positioned with a slight cephalic tilt of 15-20°, and was then positioned 10-15° contralateral oblique to visualize the greater sciatic notch, this was utilized as the trajectory view (Fig. 2). Lateral views were not able to be obtained due to patient body habitus. Once the target site was identified, using fluoroscopic guidance a sterile 22 gauge 3.5 inch spinal needle was inserted and slowly advanced until reaching the ilium, inferiorly along the sciatic notch. Advancement to this position provoked the patient's reported symptoms. Once the needle was in position, 3mL of 1 % lidocaine and 0.25 % preservative free bupivacaine and 1mL of betamethasone were injected into the area. The patient experienced >50 % relief in the subsequent 24 hours. One-month later, the patient then returned for RFA.

**Fig. 3:**
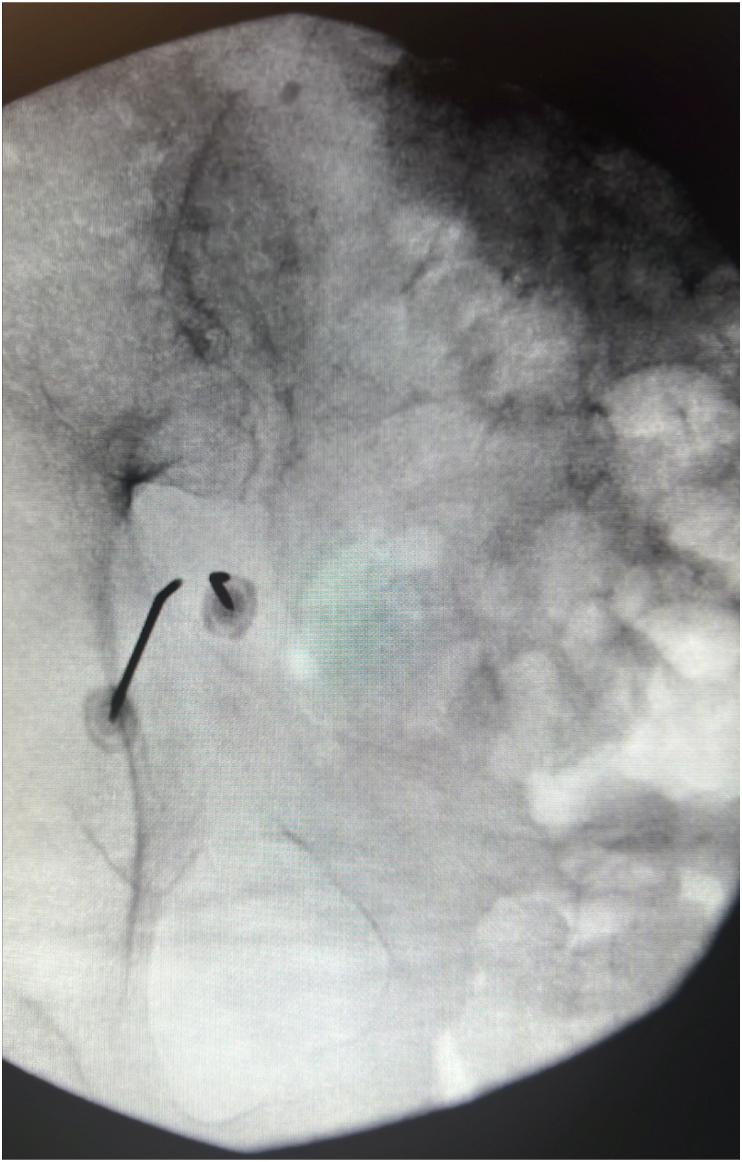
Sciatic nerve radiofrequency ablation with bipolar setting, at the sciatic notch.

**Fig. 4:**
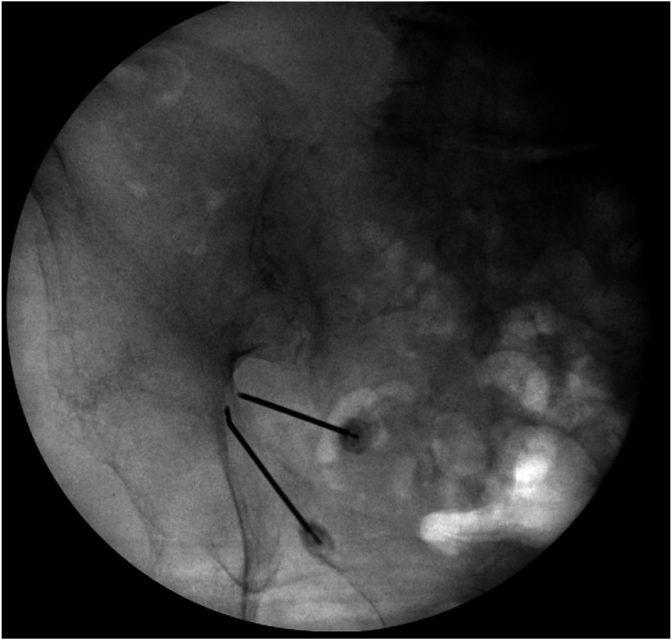
AP fluoroscopic view left hip. Radiofrequency Ablation (RFA) Left Sciatic Notch using bipolar.

Prior to the RFA procedure the patient was placed in the prone position and received local anesthetic to the skin and soft tissues of the area with 1mL of 1 % lidocaine. She did not receive sedation and was alert and responsive throughout the procedure. The fluoroscope was again positioned with a slight cephalic tilt of 15-20°, and was then positioned 10-15° contralateral oblique to visualize the greater sciatic notch, this was utilized as the trajectory view. The sciatic notch was identified and 2 sterile 17 gauge 50mm RF cannulas were positioned at the greater sciatic notch. The first cannula was advanced under fluoroscopy guidance until contacting ilium, and the second was advanced next to it (Fig. 3). Sensory testing was carried out and was found to reproduce the patient's pain. Motor testing was also carried out with 2 Hz stimulation, though the patient's residual limb was non-functional. Following this testing, 2mL of 2 % lidocaine and bupivacaine were slowly injected into the area. Following this a continuous lesion was applied for 90 seconds at 80° Celsius, with the bipolar setting to create a larger single lesion without re-positioning (Fig. 4). The patient tolerated the procedure well and released home that day.

## 4 Results

One and a half months following the sciatic nerve RFA procedure, the patient reported 80 % improvement in her symptoms. She described a decrease in frequency of phantom pain flares during the daytime, as well as improved ability to sleep throughout the night. At follow-up, her oral pain medication frequency decreased from oxycodone 5mg three times daily to 5mg once nightly. While she did report some ongoing pain, she noted less functional inhibition and overall improved quality of life.

## 5 Discussion

This novel case presents a patient with a left lower extremity hip disarticulation, whose phantom pain was exacerbated by concomitant cancer-related pain given persistent acetabulum-encasing mass involvement. She underwent RFA of the sciatic nerve at the sciatic notch after poor response to other measures. Following this procedure, she reported significant subjective improvement in her pain symptoms, and objectively decreased her opioid consumption for pain management.

In patients with lower extremity amputations, phantom limb pain is a prevalent condition which often contributes to decreased quality of life and diminished ability to perform activities of daily living [1,2,19]. Given the limitations of existing treatments researchers have begun to investigate the utility of percutaneous interventions including radiofrequency for pain relief in this patient population. Several case reports suggest peripheral nerve blocks or ablations of the sciatic or femoral nerves may provide enhanced and prolonged relief compared to medical management alone of phantom limb pain [2,3,11]^,^. Zheng et al. report a case of co-ablation of the sciatic and femoral nerves which resulted in sustained pain relief at 6 months and discontinuation of oral analgesics in a patient with a lower extremity amputation in the setting of vascular disease [3]. Other studies showed continuous block of the femoral and sciatic nerves with 0.5 % ropivacaine showed significant relief when compared to saline placebo, and a systematic review found 75-100 % decreased pain following nerve ablation at 6 month follow-up in a small case series [11,14]. Suggesting that treatment with RFA may offer more sustained pain relief for select patients.

RFA was initially developed as a minimally invasive technique which has been shown to aid in the treatment of back pain through modulating pain input to the central nervous system. It has subsequently been utilized in multiple settings to provide prolonged pain relief in a variety of conditions. RFA utilizes a high-frequency current which creates elevated temperatures, stimulation and ablation of target tissues [12]. The heat produced leads to localized destruction, and coagulative necrosis at a targeted area. This is proposed to result in degeneration of distal nerve fibers and disruption of nociceptive signals. Current research is also investigating mechanisms of molecular changes and cell signaling molecules that may also lead to improved pain relief [[12], [13], [14]]. The procedure is often described as being completed under ultrasound or fluoroscopic guidance [20]. In this case, fluoroscopic guidance was selected given less user dependence, ability to complete the desired sensory testing, as well as patient body habitus and comfort, though in certain populations ultrasound may be considered for accuracy.

In the setting of PLP, utilization of RFA may result in reduced transmission of ectopic peripheral nerve signals to the central nervous system [13,14]. Targeting of the sciatic nerve at the sciatic notch resulted in significant relief of our patient's reported residual buttock pain, and phantom knee and ankle pain. The procedure was well tolerated with both subjective and objective improvements in the patient's pain and function. Given the results of this single case study, sciatic-nerve RFA may benefit from further research and larger scale studies to investigate its safety and efficacy as a novel treatment option for hip-disarticulation patients with refractory PLP inadequately controlled with oral medications alone.

## Funding

This research did not receive any specific grant from funding agencies in the public, commercial, or not-for-profit sectors.

## Declaration of competing interest

The authors declare that they have no known competing financial interests or personal relationships that could have appeared to influence the work reported in this paper.
